# Recreational walking and perceived environmental qualities: a national map-based survey in Denmark

**DOI:** 10.1186/s12942-023-00339-2

**Published:** 2023-09-03

**Authors:** Lars Breum Christiansen, Trine Top Klein-Wengel, Sofie Koch, Jens Høyer-Kruse, Jasper Schipperijn

**Affiliations:** Department of Sports Science and Clinical Biomechanics, University of Southern Denmark, Campusvej 55, 5230 Odense M, Denmark

## Abstract

**Background:**

The aim of the study is to explore the diversity in recreational walking motives across groups with different sociodemographic characteristics, and to use a dynamic and person-centered approach to geographically assess recreational walking behavior, and preferences for place quality related to recreational walking.

**Methods:**

A total of 1838 adult respondents (age 15–90 years), who engage in recreational walking, participated in the map-based survey. We used the online platform Maptionnaire to collect georeferenced information on the respondents’ home location, other start locations for walking trips, and point of interest on their trips. Distance between home location and other start locations as well as point of interest were computed using a Geographic Information System (GIS). Additional information on recreational walking behavior and motives were collected using the traditional questionnaire function in Maptionnaire.

**Results:**

The most prevalent motives for walking were mental well-being and physical health, together with enjoyment and experiences related to walking. Having a tertiary education was positively associated with mental well-being motive, experiences, and taking the dog and the children outside. Income was also positively associated with experiences and walking the dog together with enjoyment of walking and spending time with others. Using the map-based approach, we found that recreational walking often starts at a location away from home and is not limited to the nearest neighborhood. A total of 4598 points of interest were mapped, and the most frequently reported place qualities were greenery, water, wildlife, good views, and tranquility.

**Conclusion:**

We used a dynamic and person-centered approach and thereby giving the respondents the opportunity to point to relevant locations for their walking behavior independently of their residential neighborhood. Recreational walking often starts away from home or is not limit to the nearest neighborhod. The median distance from home to the mapped points of interests was between 1.0 and 1.6 km for home-based trips and between 9.4 and 30.6 km for trips with other start locations. The most popular place quality related to the mapped points were greenery, water, wildlife, good views, and tranquility.

**Supplementary Information:**

The online version contains supplementary material available at 10.1186/s12942-023-00339-2.

## Introduction

Walking is the most common physical activity (PA) in everyday life across most population groups, regardless of age, social class, race, or gender [1, 2]. It is an easily accessible activity that does not require special skills, infrastructure, or equipment [1]. Walking can be done with the purpose of transportation to a given destination, or as part of recreation. Walking for recreation is multifaceted behavior consisting of many forms and motives, such as exercise, experiences, adventure, social relations, mental and physical health. When walking, people demonstrate various levels of commitment, from occasional strolling to more challenging physical experiences on a more frequent and committed basis [3]. Still, little empirical evidence exists that describes the prevalence and distribution of these motives, which can provide important information for the stakeholders involved in the supply and management of walking resources [3].

Following the socioecological model, health behavior is a complex behavior influenced by factors at different levels, often including the build environment to which individuals are exposed, interpersonal characteristics, and intrapersonal characteristics [4, 5]. Sociodemographic differences exist in walking behavior and as an example evidence suggests that individuals with a shorter education and lower income are less affected by a supportive walking environment [6]. In a recent review, Hilland et al. [7] found that the most consistent correlates of recreational walking for socioeconomically disadvantaged adults were perceived neighborhood aesthetics, social support from friends and family and perceived safety. Differences in recreational walking across sociodemographic groups might therefore be explained by several interrelated factors at various levels.

The interaction with the built and natural environment is a central part of recreational walking, but little is known about walking and its relation to route-choice and place quality. Some people have a strong motivation for the activity itself, while others are more motivated by the routes and locations [3]. Previous studies found associations between recreational walking and different environmental characteristics including land use mix, street connectivity, availability of destinations, access to facilities (e.g., bench, table, toilet), traffic-related safety, crime-related safety, route quality, aesthetics, and attractiveness [8–19].

The reviews by Sugiyama et al. [13] and Hilland et al. [7] underline the importance of aesthetics and perceived quality of recreational settings for recreational walking as it was found more important than the sole presence of green areas. These reviews call for more research in this area including an exploration of novel methods for measuring relevant qualities, which is in line with a review by Calogiuri and Chroni [20]. With a focus on the natural environments’ role in supporting motivation for active living, the review emphasized the role of views of parks, gardens and nature. Recently, Mondal et al. [21] investigated emotional states during walking episodes and highlighted the importance of pleasant environments and their relation to positive emotions. Perceived qualities or values in the environment reflect a variety of elements that are important to the individual [22].

Capturing or measuring exposure to the built and natural environment is not straightforward. Some studies have used objective measures of the environment using Geographic Information Systems (GIS), others have used perceived self-reported measures, and others have used visual systematic assessment of e.g., park and street qualities [23]. Many studies have used the residential environment or neighborhood, which has raised the discussion on how to define individuals’ spatial exposure [24]. For example, recreational walking can be undertaken from home and in the near vicinity of home, but individuals may also visit other places further from home, for different reasons. A more dynamic and person-centered approach has been developed that uses an online map-based survey to collect subjective data from participants. These methods, sometimes referred to as SoftGIS or Public Participation GIS (PPGIS), have been used to involve the public in city planning perspectives as well as in research studies [22, 24, 25]. In short, the geospatial technology consists of an online map-based survey, where respondents are asked to place markers or draw line or areas on a digital map representing behaviors, experiences or attitudes to specific locations. Altogether, recreational walking is easily accessible for most people, and has documented positive effects on both physical and mental health. Differences in walking behavior across sociodemographic groups are not well understood and specific knowledge on differences in motives and preferences is needed to inform future interventions.

The aim of the study is to explore the diversity in recreational walking motives across groups with different sociodemographic characteristics, and to use a dynamic and person-centered approach to geographically assess recreational walking behavior, and preferences for place quality related to recreational walking.

The aim of the study is to investigate and understand the variations in recreational walking motives among different groups with diverse sociodemographic characteristics such as age, gender, socioeconomic status, and other relevant demographic variables. The study employs a dynamic and person-centered approach to geographically assess recreational walking behavior. This means that the study will consider the specific locations where individuals engage in recreational walking, considering factors such as accessibility, aesthetics, and environmental quality. The study seeks to understand individuals' preferences for place quality related to recreational walking. Place quality refers to the overall attributes and features of a particular location that contribute to its desirability for recreational walking. By exploring walking behaviors and place quality preferences, the study aims to identify the key factors that individuals consider important when choosing a place for recreational walking.

## Methods

### Study design—Moving Denmark and original survey

This study is a part of the research project ‘Moving Denmark’ which examines physical activity and sport participation among the adult Danish population (15 years and older) using surveys, qualitative interviews, and device-based measurements. The research project’s overall focus is to assess the prevalence of physical active behaviors across all 98 municipalities in Denmark and to explore the relationship between opportunities and motives for a broad variety of physical activities across groups with different sociodemographic backgrounds. This knowledge should be applicable and usable for the development of policies and design targeted initiatives with the aim of increasing the level of physical activity and sports participation by Danish people.

The current study is a sub-study of ‘Moving Denmark’, and the participants were recruited from respondents of the original national survey. The original survey was conducted in Denmark between October 19th and November 29th, 2020. A total of 404,000 adult Danes (< 15 years) from municipalities throughout the country were invited mainly through a national online digital mailbox system (e-boks). Two reminders were sent, and eight prizes of 1.300 EURO were drawn from completing respondents. A total of 157,858 respondents answered the survey which corresponds to a response rate of 39%. The questionnaire contained questions regarding citizens’ physical activity habits, motives for being active, settings and opportunities for PA and their background (education, work etc.). The questionnaire also contained a section where they could consent to being contacted again for participation in sub-studies including the map-based survey for the study presented in this paper.

### Participants in map-based questionnaire

Inclusion criteria for participation in the current study of recreational walking were a positive consent to subsequently receive an invitation to the map-based survey and to have reported recreational walking at least once a month during the last year. Of the eligible potential participants (56.505 respondents) a random sample of 8.000 persons throughout the country was drawn to receive an invitation to participate. The sample size was based on practical as well as economic reasons and to ensure sufficient data for subgroup analyses.

### Structure and content of the Map-based survey

The online map-based survey was created and distributed using Maptionnaire (https://maptionnaire.com/). The map-based survey enables a dynamic and person-centered approach and contains the opportunity to geographically assess recreational walking behavior. The respondents were presented with a Google map with satellite imagery zoomed to their municipality. From this point they were able to zoom in and out to identify specific locations, place markers and answer geographically related questions. Additionally, they answered general questions related to walking behavior and motives for walking. The questionnaire was extensively tested for understanding and functionality by the authors, experts, and lay persons before data collection.

- *Walking behavior:* The questions regarding respondents’ recreative walking behavior were divided between home-based trips and trips with another start location. Information on frequency, duration, social company, and use of step-counter device were collected.
- *Motives*: This question contained 11 possible reasons for walking e.g. “*I walk during my leisure time for my physical health”* (cf. Table 2)*.* Respondents answered on a five-point Likert scale from totally agree to totally disagree. The 11 motives were compiled and selected based on previous research on general and specific motives for physical activity and recreational walking [25–27].
- *Point of departure for trips:* Respondents were asked to place a marker at a digital map at their home address and at other departure locations for recreational walking trips within the last month. They could place as many markers as they wanted. For makers placed for other departure points than home they were asked for their means of transportation to this location.
- *Point of interest along the walking routes:* For walking trips during the last month departing from home and from other places, respectively, the respondents were asked to place markers on the digital map for locations they experienced as a “good location”. For each marker they would get a follow-up question regarding the characteristic of that location. From a list of 21 qualities, they could select one or several, and they could also choose the category *“other”,* which was then followed by a free text description (cf. Table 3). The 21 qualities/values were selected and compiled based on previous Danish and international studies and frameworks related to landscape values and preferences for recreational and walking experiences [9, 22, 25, 26, 28, 29].

### Data collection

Invitations to the map-based survey were sent out in May 2021 to the 8000 selected participants. At that time, all shops, services, and sport facilities were open, but face masks were still required indoors, and most of the population had initiated the COVID-19 vaccination program. The invitation with a link to the survey was sent out by regular e-mail, which the participants had provided in the original survey. The survey took 15–30 min to complete. One reminder was sent one week after the invitation and the data collection ended mid-June 2021. A total of 289 e-mails could not be delivered and 56 participants chose to withdraw from the study for various reasons. Of those, 18 reported they had withdrawn due to technical issues with the map-based survey.

### Sociodemographic variables

Sociodemographic variables were obtained from Statistics Denmark using the Danish Civil Registration System, which compiles personal information for each citizen in Denmark [30]. The data from the map-based questionnaire were merged with the following variables from Statistics Denmark: age, gender, education, equivalized disposable income [31] and place of residence. Education was dichotomized to having a tertiary education or not. Equivalized disposable income (total disposable income equivalized by household size) was converted in units of 1000 EURO. To accommodate for outliers, income was truncated to ± 2SD from mean (180 individuals). Age was converted into units of 10 years and place of residence was dichotomized to living in a city with more than 50.000 inhabitants or not.

### GIS and statistical analysis

The map-based survey contained the following geolocated variables: points for home addresses, points for other departure locations, points for qualities/values related to trips from home and for trips with other start locations. These points were transferred to ArcGIS Pro and following two variables were created: (1) Euclidian distance from home point to each point for other start locations and (2) Euclidian distance from home point to each point of interest regardless of the location of departure. The final dataset was based on georeferenced points linked to the other personal variables for each respondent. Each respondent could appear with multiple points in the dataset.

Besides ordinary descriptive statistics including prevalence, frequency and averages, logistic regression models were used to analyze the association between motives for walking, qualities for the point of interest, and sociodemographic variables. For the logistic regression on motives the response categories were dichotomized to contain totally agree and agree or opposite totally disagree, disagree or neither. For the logistic regression on qualities of the point of interest, data were analyzed at point level including random-effects at the individual level (several points per respondent). All statistical analyses were conducted using Stata 17.0.

## Results

Out of the 404,000 invited participants in the original survey 157,858 respondents answered the questions relevant for this study. In this population 90.0% marked that they had done some recreational walking in the last 12 months and 74.6% indicated that they had done it at least once a week (Table 1). In the current study 1838 respondents participated out of the 8000 invited randomly selected participants who had accepted to be invited in the original survey. The respondents in our sample were older (54.3 years vs. 51.4 years), more often female (60.5% vs. 54.8%), more often with a tertiary education (85.4% vs. 74.7%) and had a higher income equivalent per year (49,508 € vs. 43,751 €). Of the 67.6% who reported using a step counter-device 85.8% estimated their daily average to be above 6000 steps and 43.9% to be above 10,000 steps (Table 1). Relatively more females reported using a step counter-device (71.7% vs. 61.5%) and the most common device was a smartphone application (32.6%) followed by using wrist worn device (28.5%).

**Table 1:** Descriptive statistics of participants’ walking behavior and sociodemographic characteristics in the original survey and in the subsample participating in the map-based survey on recreational walking

	Large survey (n = 157,858)	Map-based survey (n = 1838)
Recreational walking (12 months)	90.0%	100%
Weekly walking (12 months)	74.6%	89.0%
Age	51.4 years	54.3 years
Female	54.8%	60.5%
Proportion with tertiary education	74.7%	85.4%
Income equivalent	43,751 €	49,508 €
Proportion living in the city (> 50,000)	38.1%	39.2%
User of step counter-device	–	67.6%
> 10,000 steps per day (n = 1070)		43.9%

Table 2 presents the prevalence of the general motives for walking and the association with sociodemographic characteristics. Four motives for walking were the clearly most prevalent: for my physical health (fully agree/agree: 92.5%), for my mental well-being (fully agree/agree: 87.1%), to experience something (fully agree/agree: 86.6%) and I enjoy walking (fully agree/agree: 90.0%).

**Table 2:** Prevalence of respondents’ motives for recreational walking and the association with sociodemographic characteristics (n = 1838)

		Prevalence (Agree or fully agree) (%)	Odds ratio with sociodemographic characteristics^#^				
			Tertiary education (**yes**/no)	Higher income equivalent (1000 €)	Older age (10 years)	Females (**yes**/no)	City dweller (**yes**/no)
Health	For my physical health	92.5%	1.24	0.98	1.45***	2.68***	0.89
	For my mental well-being	87.1%	1.72**	1.06	0.86**	2.54***	0.95
Activity related	I enjoy walking	90.0%	1.02	1.11*	1.12*	2.49***	1.03
	To experience something (e.g., people, nature, places)	86.6%	1.48*	1.08*	0.91*	1.41*	0.92
	To prepare for longer walking trips or events	19.0%	1.22	1.02	1.05	1.16	0.92
Social and solitary	To spend time with others	56.2%	1.26	1.09**	0.81***	1.32**	1.39**
	Others ask me to come	51.1%	1.15	1.06 *	0.84***	1.67***	1.40**
	To spend time alone	45.2%	1.11	1.02	0.69***	1.72***	0.93
Children and dogs	Walk the dog	26.9%	1.31*	1.06*	0.93*	1.00	0.74**
	Follow my children outside	18.0%	3.05***	1.03	0.62***	1.15	0.94
	Walk with baby carriage	6.0%	2.30*	0.98	0.69***	1.12	1.27

^#^: Response categories were dichotomized to contain totally agree and agree or opposite totally disagree, disagree or neither, the latter being the reference group. Bold values: significant OR; *: p < 0.05, **:p < 0.01 and ***: p < 0.0001

The odds ratio for agreeing or fully agreeing with each motive were associated with multiple sociodemographic variables. Having a tertiary education was associated with the mental well-being motive as well as to experience something and follow children outside. Higher income was also associated with the motive of experiencing something. Additionally, it was associated with enjoyment, walking the dog and social motives i.e., to spend time with others and being invited.

Age was significantly associated with the motives: physical health and enjoying walking. It was inversely associated with eight other motives among others: mental well-being, spend time alone and spend time with others. Women were also more likely to state to agree on several motives. This included mental and physical health, enjoyment and experience as well as the social motives and spending time alone. Finally, living in a larger city (> 50,000 inhabitants) was associated with the social* motives* and inversely associated with walking the dog.

**Fig. 1:**
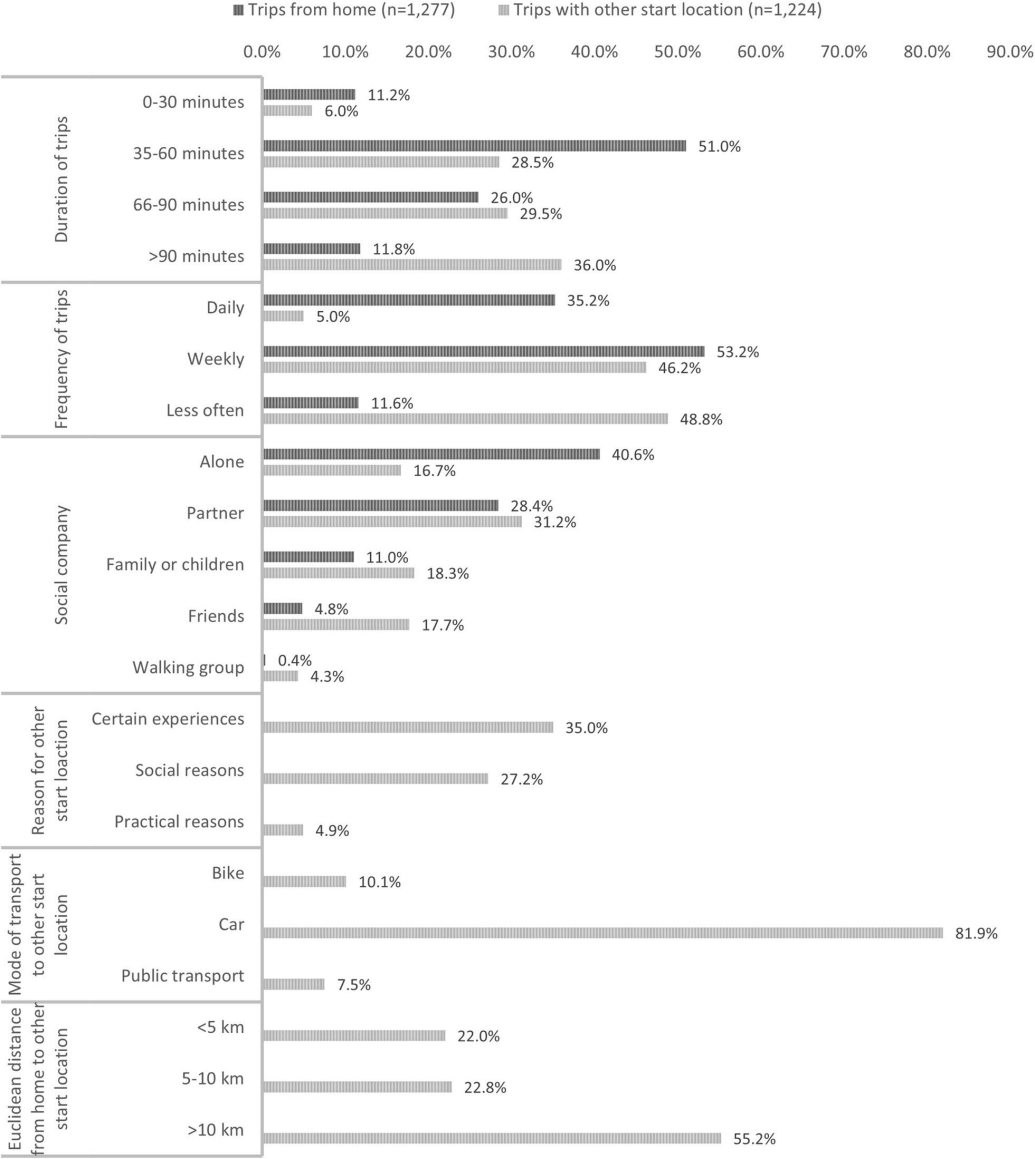
Description of walking behavior for trips with departure from home and with other start locations

Figure 1 presents the descriptive analyses of the respondents’ walking behavior and their mapping out home location and other starting locations. Even though 95.4% and 71.4% of the respondents reported to have had walking trips from home and other places, respectively, only 71.0% and 44.0% mapped a location. The pop-up questions for these mapped starting locations revealed that trips lasting 60 min or less were more prevalent for home-based trips, while 36% of trips with other start locations lasted more than 90 min. Trips from home were more likely to take place daily (35.2% vs. 5.0%) and weekly (53.2% vs. 46.2%) than trips from other places.

The trips from home were most often walked alone (40.6%) or with a partner (28.4%). For other trips, a partner was the most common company (31.2%), while friends, family and walking groups were more likely to join during these trips than for trips from home. To meet with others was also reported as a reason for another start location by 27.2% of respondents, which underlines the social factor for these walking trips. The most stated reason for other start location was to get certain experiences during the walk. A total of 10.1% cycled to the start location, 81.9% drove by car while 7.5% went by public transport. Approximately half of the other start locations were more than 10 km from home and 22% were closer than 5 km. Some start locations were very far from home and optional open text responses revealed that walking trips during vacations, weekends in the summer house, and social family gatherings were a common explanation.

**Fig. 2:**
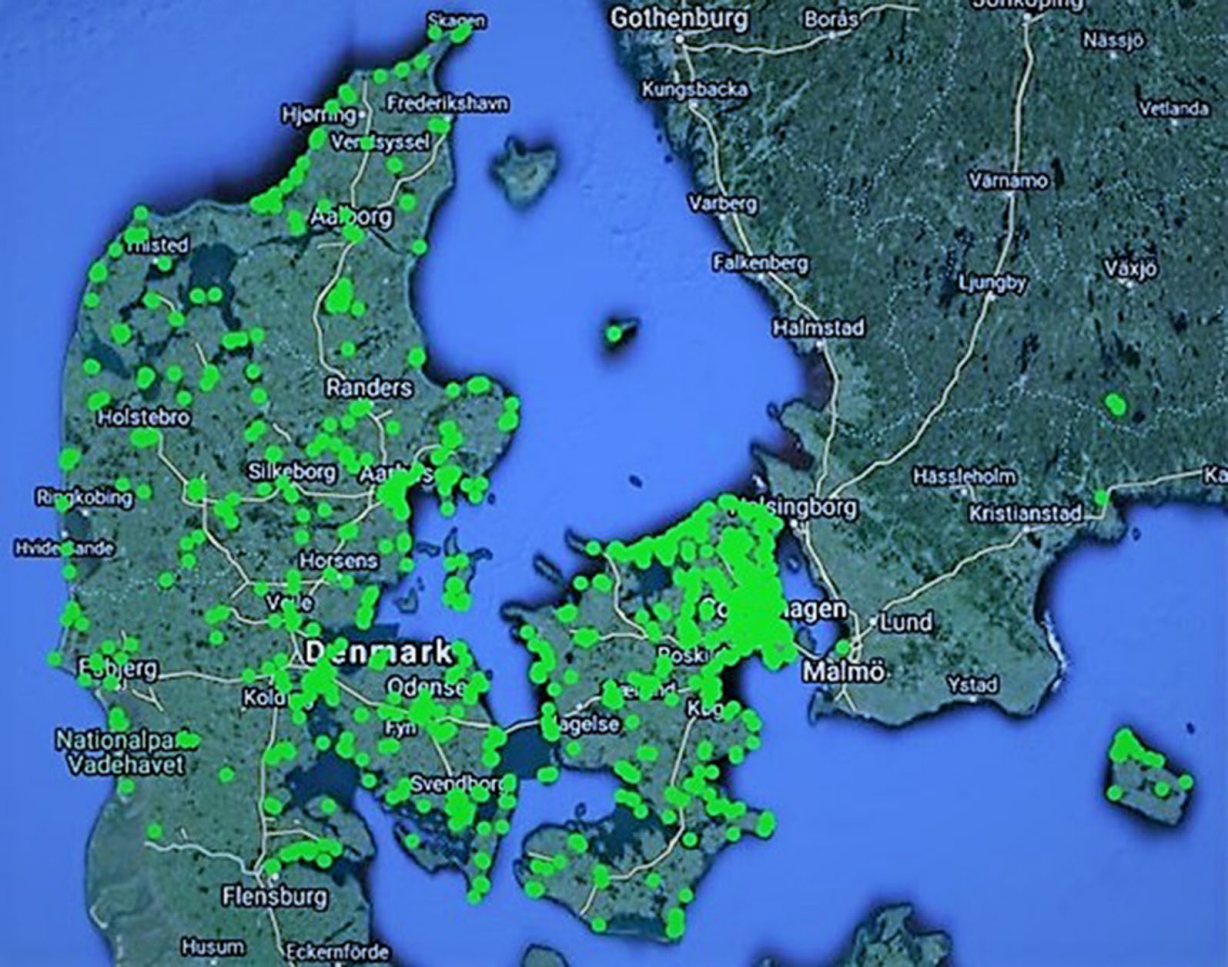
Visualization of point of interest for the participants

Finally, we assessed the preferences for place quality related to recreational walking trips, their distance from home, and relationship with sociodemographic characteristics. Of the 1838 respondents to the map-based survey, 42.2% and 74.7% did not place any markers for points of interest (POI) for home-based trips or trips with other start locations, respectively. The remaining 1062 respondents on average mapped 3.3 POI related to home-based trips and a total of 465 respondents mapped in average 2.3 POI related to other start locations. For the home-based trips, 8.1% of the POI had no place quality characteristic attached, 30.9% had 1–3 qualities, and 61% had 4 or more qualities. For the trips with other start location, 9.3% had no place quality characteristic attached, 24.4% had 1–3 qualities and 66.4% has 4 or more qualities (data not shown). Figure 2 visualizes the geographic distribution of points across Denmark.

The prevalence of place quality characteristics selected as POI are presented in Table 3 together with the median distance from the POI to the mapped home point. In the last column, the odds ratio for a characteristic to be selected by a respondent from different sociodemographic groups and start location are presented. More detailed results of the logistic regression analysis can be found in Additional file 1: Table S1.

**Table 3:** Descriptive statistics and sociodemographic association of quality characteristics of the mapped point of interest related to trips from home and other start locations

	Quality characteristics of the mapped point of interest	Prevalence of quality of total points (%)		Median distance from home (km)		Association^a^
		Trips from home (n = 3528)	Trips with other start location (n = 1070)	Trips from home (n = 2654)	Trips with other start location (n = 774)	
Aesthetics/scenic	Greenery (e.g., trees, flowers)	72.4%	72.2%	1.2	12.7	Female ( +)
	Water	44.1%	55.6%	1.3	15.5	Age ( +), income ( +), city dweller ( +), other start location ( +)
	Wildlife	41.2%	49.2%	1.3	14.1	Other start location ( +)
	Attractive view	47.1%	56.6%	1.4	14.3	Age ( +), income ( +), city dweller (−), other start location ( +)
	Attractive buildings	10.0%	9.2%	1.3	12.9	Age (−), tertiary education (−), city dweller ( +)
	Historic surroundings (e.g., buildings and neighborhoods)	9.8%	11.4%	1.5	21.4	Other start location ( +)
Functional/service	Food and drinks	5.2%	6.9%	1.5	16.3	Tertiary education (−), city dweller ( +), other start location ( +)
	Playground/activity area	6.3%	3.8%	1.0	9.4	Age (−)
	Benches/picnic areas	16.1%	16.4%	1.2	11.1	
	Toilet	5.1%	7.2%	1.6	16.7	Other start location ( +)
	Well-lit	4.4%	2.6%	1.0	30.6	Tertiary education (−)
Path-/route-quality	Plain terrain	27.8%*	21.3%	1.1	13.8	Age (−), other start location (−)
	Hilly terrain	24.6%	31.4%	1.3	17.8	City dweller (−), other start location ( +)
	Wide paths to go side-by-side	27.5%	27.5%	1.2	11.6	Age (−)
	Even surface	24.8%	23.4%	1.2	11.4	Female ( +)
	Challenging route	8.0%	12.7%	1.5	21.5	City dweller (−), other start location ( +)
Social-/personal atmosphere	Tranquility	40.6%	41.1%	1.2	14.2	Age (−)
	Solitude	17.2%	13.6%	1.3	12.7	Age (−)
	Meet people	15.9%	14.1%	1.1	14.5	Age (−), tertiary education (−)
	Personal significance	8.7%	12.1%	1.3	25.7	Other start location ( +)
	Good atmosphere	29.5%	29.8%	1.3	12.7	Age (−), female ( +), other start location ( +)

a: Association between quality characteristics of the mapped point of interest with sociodemographic characteristics as well as start location for the trip. “( +)” indicates a significant OR > 1 and “(−)“ indicates a significant OR < 1. For full logistic regression analyses see Additional file 1: Table S1

In the group of aesthetic/scenic characteristics, *greenery* was most often selected with more than 70% of the POI for both home-based trips and trips from other locations. The median distance from home to POI with these characteristics was 1.2 km for home-based trips and 12.7 km for other trips. Water, wildlife, and attractive view were selected for between 41.2% and 56.6% of POIs and were more likely to occur at a POI with other start locations. Historic surrounding and attractive buildings were the least prevalent, but still selected for approximately 10% of the POI.

The group of functional/service characteristics were less often selected as a quality. Benches and picnic areas were most common with 16.1–16.4%, for home and other starting locations respectively, while toilet and food/drinks were more often selected for POI with other start points. Playground and well-lit places were the POI characteristics with the shortest median distance from home (1 km).

Plain terrain, hilly terrain, wide paths and even surfaces were selected for between 21.3% and 31.4% of POI. A challenging route was less popular with 8.0% and 12.7%, respectively. Hilly terrain and a challenging route were more often selected for a POI with another start location and with 17.8 km and 21.5 km, these POI had some of the longest median distances from home.

In the final category, tranquility was the most common characteristic with a prevalence at 40.6% and 41.1%, respectively. The opposites, solitude and to meet people were evenly popular with selection prevalence at between 13.6% and 17.2%. POI with personal significance were the least common, but were more often selected for other start locations and had one of the longest median distances with 25.7 km. Finally, good atmosphere was selected for approximately one third of the POI.

Differences between sociodemographic subgroups were significant for most characteristics in the multilevel logistic regression models. Higher income was associated with higher odds ratio for water and attractive views, while respondents with a tertiary education were less likely to have selected attractive buildings, food and drinks, well-lit and the possibility to meet people. Females were significantly more likely to have selected greenery, even surface and good atmosphere. Older age was associated with higher odds ratio for water and attractive views, but lower odds ratio for attractive buildings, playgrounds, plain terrain, wide paths, tranquility, solitude, possibility to meet people and good atmosphere. Finally, respondents living in larger cities with more than 50,000 inhabitants more often selected water, attractive buildings and food and drinks, but less often attractive view, hilly terrain and challenging route.

## Discussion

We aimed to explore the diversity in recreational walking motives across groups with different sociodemographic characteristics, and to use a dynamic and person-centered approach to geographically assess recreational walking behavior, and preferences for place quality related to recreational walking. The discussion will take its starting point in the three most dominant motives for recreational walking: health, experience, and social motives. It is followed by a discussion of the map-based approach to assess recreational walking behavior and related methodological reflections.

### Recreational walking motives across groups

The analysis of motives for recreational walking showed that the physical and mental well-being was almost equally important for the respondents. The focus on the physical health benefits was underlined by the large share (67.6%) of respondents using a step counter-device and the motive was most prevalent for women and increased with age. This is not a surprising finding, as personal factors, or what have been called push factors (3), are important for walking behaviors. One of these factors is the motive for exercise [29]. The focus on mental health has gained more attention during the last decade, and a review of the evidence of walking suggest there is substantial evidence for positive effects on anxiety and depression and limited but emerging evidence for the positive effect on psychological stress and psychological well-being [32]. Having a tertiary education, being younger, and female were associated with the mental well-being motive.

Even though health and well-being are strong motives for recreational walking, a large majority also selected to enjoy walking and to experience something as their motives. Walking with the purpose of gaining a certain experience is what has been called pull factors [3], which is a key connection between recreational walking and environmental qualities. The experience motive was selected by 86.6% of the respondents and associated with being younger and female in addition to having a tertiary education and a higher income. To *enjoy walking* might be related to environmental qualities as well as other perceived benefits or internal values. This motive was also associated with higher income and being female, as well as older age, but not tertiary education. These findings are in contrast to the study on emotions during walking, who concluded that higher income and education were associated with less positive emotions during recreational walking [21]. Mondal and colleagues suggested that this might be due to excessive workload and time constraints in this part of the population [21]. The study methods were not similar, but an explanation for our differing findings could be the possibility for a break from a stressed day, a higher appreciation for environmental qualities, or more pleasant environments to walk.

Recreational walking often involves a social motive and is done in the company of others in 59.4% of trips from home and in 83,3% of trips with other start locations. The social motives to spend time with others and others ask me to come were more prevalent among women, younger individuals, city dwellers and those with higher income. Women were not more likely to select the care-giver motives of taking dogs, children or baby carriages for a walk. However, this was more prevalent for younger individuals and for individuals with a tertiary education. The association with age in relation to children is logical and aligned with the inverse association between selecting a playground as an environmental quality. The relation to tertiary education is not as straight forward. It could be explained by differences in values about childcare and the importance of outdoor experiences for their children. Another explanation could be a higher focus on risk, or simply a difference in parental status across this group.

### Recreational walking behavior, and preferences for place qualities

Recreational walking is the most common physical activity for many people, and our results show that a variety of environmental characteristics, in combination with a range of personal factors, are predictive for recreational walking. Our results also show that it is not just the local neighborhood that is important, but a much larger geographical area. Other studies of environmental characteristics have used a neighborhood-based approach with different buffers around the home address to assess spatial exposure, but in this study, we used a dynamic and person-centered approach. The nearest environment within, for example, 1 km from home might have importance for recreational walking behavior, but our study shows that 71.4% of the recreational walkers use other start locations for their trips, and that the median distance to POIs is more than 1 km away from the home address. This is in line with, for example, Kajosaari and Laatikainen [33], who found that 40% of visits to leisure time physical activity locations were placed more than 1600 m from home, specifically for public green space, it was 33.6% of visits. Other studies have found consistent relationships between recreational walking and neighborhood-centric built environment measures [13, 15, 16]. Kajosaari and Laatikainen [33] points to another important aspect, which is that the least physical active participants tended to concentrate their activity closer to home. Our study participants are likely to more motivated and more active recreational walkers, and therefore have a wider range of destinations and use areas further from home.

In this study, we focused on recreational walking, particularly its relationship with perceived environmental qualities. Previous research has argued that recreational walking is less dependent on the environment compared to transport-related walking, as it does not require a specific destination [6]. However, based on our results, we would argue for the opposite since experience and enjoyment of the walking trip were prevalent motives. Aesthetic and scenic qualities, as well as atmospheric qualities, were among the most selected for the POI’s, which is in line with Mirzaei et al. [15] argument, that these qualities are crucial due to their relation to intrinsic motivation. We found that the mental well-being motive was nearly as common as the physical health motive, which underlines the importance of place qualities. Place qualities could foster mental well-being in several ways, with tranquility, solitude, and good atmosphere, being the more obvious ones. These qualities were also selected for a larger proportion of the POI’s.

Previous research has found a positive association between recreational walking and the presence of destinations and attributes of recreational destinations, such as parks [13]. Furthermore, the review highlighted that this association was particularly strong for the studies which included the quality of recreational destinations and route aesthetics. Hilland et al. [7] reinforced the significance of perceived neighborhood aesthetics and emphasized the importance of subjective perceptions of walkability and individual safety in influencing walking behaviors, especially among disadvantaged groups. The current study did not include environmental barriers like safety, fear of crime, traffic, noise, poor aesthetics which all can impact walking behaviors. The person centered SoftGIS approach used in the current study has previously been utilized to collect both positive and negative qualities related to the environment [34]. When implemented in the Helsinki metropolitan area and not focused on recreational walking, the top three positive qualities were attractive surroundings, possibilities for cycling and walking, and the presence of nature. The top three negative qualities were unattractive surroundings, a hectic environment and a feeling of social insecurity [34]. This study’s findings align with the emphasis on subjective perceptions of the environment in the reviews. While the study identified specific characteristics like greenery, attractive views, and tranquility as commonly selected, it underscores that subjective perceptions of walkability and neighborhood aesthetics are crucial in shaping walking behaviors.

### Methodological reflections

The study invited a random subsample of consenting individuals who had participated in a nationwide survey in which they stated they engage in recreational walking. The sampling method increases the generalizability of the findings to Denmark and similar countries, encompassing both urban and rural areas, as well as areas and municipalities with different sociodemographic compositions. The study only includes respondents who reported to engage in recreational walking, and as such, we can only draw inferences regarding their motives and actual preferences and the differences among them. In other words, we had no data on people who do not walk for recreation. The large number of respondents and many POI’s across the country contribute equally to the generalizability of the results. However, the broad study area (an entire country) resulted in a low concentration of respondents in specific geographic regions and limited the use of certain spatial analysis methods, compared to more focused study regions. For example, in the current study, we were unable to investigate POIs in the same city across different socioeconomic groups due to the limited number of respondents in each city.

This study applied SoftGIS methods without limiting respondents to where they could place their walking trip start points and POIs. Many other studies of the relationship between physical activity and environmental characteristics are neighborhood-centered using census districts or home-based buffers with radii of 500 m or 1 km. However, in this study most of the POI were placed further away than 1 km from home. This was true for both trips from home and those starting from other locations, showing that people use a (much) larger ‘neighborhood’ when they walk for recreation. At the same time, map-based surveys are very practical for collecting empirical information on the subjective environment and perceived quality on the walking routes.

The original survey and the current study were conducted during the COVID-19 pandemic, which affected physical activity throughout the world [35]. While the data collection period for the current study saw the reopening of shops, services, and sport facilities, and the pandemic was on decline in Denmark, it is possible that the pandemic affected some of the results. For instance, it may have impacted the popularity of some of the environmental qualities: tranquility, solitude, and meeting other people, as well as the priority of spending time in the nature in general [36].

This study applied an exploratory approach and analyzed several associations between sociodemographic variables and a range of motives and environmental qualities. Therefore, multiple testing is a concern and should be considered in the interpretation of the results. Furthermore, as this is an exploratory study, further research is needed to draw conclusions on the causal relations between walking for recreation and environmental characteristics, as well interventions to promote recreational walking.

## Conclusions

This study used a map-based survey to explore recreational walking among 1838 adults. We found that the most prevalent motives for walking were mental well-being and physical health, together with enjoyment and experiences (people, nature, places) related to walking. All four motives were more prevalent among women. Well-being and experiences were more prevalent among those with a tertiary education and younger age, enjoyment and experience were positively related to higher income, while physical health and enjoyment were positively related to older age.

We took a dynamic and person-centered approach by using a map-based survey and thereby giving the respondents the opportunity to point to relevant locations for their walking behavior independently of their residential neighborhood. Recreational walking often starts away from home or is not limit to the nearest neighborhood. A total of 4598 points of interest were mapped related to trips from home and trips with other start locations. The median distance from home to the mapped points was between 1.0 and 1.6 km for home-based trips and between 9.4 and 30.6 km for trips with other start locations. The most popular place quality related to the mapped points were greenery, water, wildlife, good views, and tranquility.

### Supplementary Information

**Additional file 1: Table S1. **Association between points of interest’ quality characteristics and sociodemographic characteristics as well as point type (the odds ratio for the POI to have a specific characteristic). Presented overall direction and significance in Table 3.

## Data Availability

The datasets used and/or analyzed during the current study are available from the corresponding author on reasonable request.
