# Supplementary material for: Recreational walking and perceived environmental qualities: a national map-based survey in Denmark
Source: Int J Health Geogr. 2023 Sep 3;22:21. doi: 10.1186/s12942-023-00339-2 (PMC10476396; doi:10.1186/s12942-023-00339-2)
Supplement: Supplementary file 1 — Additional file 1: Table S1. Association between points of interest’ quality characteristics and sociodemographic characteristics as well as point type (the odds ratio for the POI to have a specific characteristic). Presented overall direction and significance in Table 3. [file 12942_2023_339_MOESM1_ESM.docx]

**Additional file 1: Table S1** Association between points of interest’ quality characteristics and sociodemographic characteristics as well as point type (the odds ratio for the POI to have a specific characteristic). Presented overall direction and significance in Table 3

|  | **Quality characteristics of the mapped point of interest** | Odds ratio with sociodemographic characteristics^#^ | | | | | |
| --- | --- | --- | --- | --- | --- | --- | --- |
|  |  | Point type  (**Other**/ home) | Older age  (10 years) | Females  (**yes**/no) | Tertiary education  (**yes**/no) | Higher income equivalent  (1000 €) | City dweller  (**yes**/no) |
| Aesthetics/scenic | Greenery (e.g., trees, flowers) | 1.03 | 1.02 | 1.54** | 1.13 | 1.00 | 1.09 |
|  | Water | 2.04*** | 1.09** | 1.06 | 1.00 | 1.07** | 1.23* |
|  | Wildlife | 1.79*** | 1.09 | 1.06 | 1.40 | 1.06 | 1.05 |
|  | Good view | 1.93*** | 1.10* | 1.09 | 0.93 | 1.07* | 0.75* |
|  | Attractive buildings | 1.03 | 0.87** | 0.85 | 0.51** | 1.00 | 1.65** |
|  | Historic surroundings (e.g., buildings and neighborhoods) | 1.33* | 1.03 | 0.78 | 0.77 | 0.96 | 1.04 |
| Functional/service | Food and drinks | 1.64** | 0.92 | 1.29 | 0.42*** | 0.94 | 1.64** |
|  | Playground/activity area | 0.73 | 0.72*** | 1.35 | 0.87 | 1.01 | 1.29 |
|  | Benches/picnic areas | 1.25 | 0.91 | 1.07 | 0.80 | 0.94 | 1.27 |
|  | Toilet | 1.99*** | 1.03 | 1.14 | 0.70 | 0.96 | 1.05 |
|  | Well-lit | 0.77 | 0.74 | 1.73 | 0.33* | 0.80 | 1.75 |
| Path-/route-quality | Plain terrain | 0.61*** | 0.85** | 0.87 | 0.65 | 0.95 | 1.36 |
|  | Hilly terrain | 1.77*** | 0.98 | 1.15 | 1.36 | 1.04 | 0.53*** |
|  | Wide paths to go side-by-side | 1.18 | 0.79* | 1.25 | 0.69 | 0.97 | 1.12 |
|  | Even surface | 0.94 | 0.99 | 1.50* | 0.65 | 0.91 | 1.28 |
|  | Challenging route | 1.95*** | 0.99 | 0.85 | 1.16 | 0.97 | 0.49** |
| Social-/personal atmosphere | Tranquility | 1.14 | 0.82*** | 1.20 | 1.04 | 0.97 | 0.74 |
|  | Solitude | 0.75 | 0.64*** | 1.46 | 1.17 | 0.94 | 0.68 |
|  | Meet people | 1.13 | 0.81** | 1.08 | 0.55*** | 0.98 | 1.25 |
|  | Personal significance | 1.70** | 1.02 | 1.18 | 1.19 | 0.94 | 0.83 |
|  | Good atmosphere | 1.51** | 0.67*** | 1.55* | 0.75 | 0.92 | 1.40 |

#: Each POI are dichotomized to have a specific characteristic or not, the latter being the reference group. *: p<0.05, **:p<0.01 and ***: p<0.0001
